# Relationship between parental physical activity and adolescents’ exercise cognition: the mediating role of family activity support

**DOI:** 10.3389/fpubh.2025.1685991

**Published:** 2025-12-02

**Authors:** Fusheng Liang, Huasen Yu, Feifei Li, Xingying Li

**Affiliations:** 1 School of Physical Education and Health, East China Normal University, Shanghai, China; 2 School of Physical Education, Huangshan University, Huangshan, Anhui, China; 3 School of Physical Education and Health, Yili Normal University, Yining, China

**Keywords:** parental physical activity, family activity support, adolescents, exercise cognition, cross-sectional study

## Abstract

**Background:**

Adolescents’ exercise cognition influences their healthy development. While parental physical activity (PA) is linked to Adolescents’ exercise cognition, the role of family activity support in this relationship is still unclear. This study aims to examine how family activity support influences the relationship between parental PA and adolescents’ exercise cognition.

**Methods:**

A 2023 survey conducted across 15 provinces in China ultimately included 12,457 participants. Multiple linear regression examined the links between parental PA, family activity support, and adolescents’ exercise cognition, adjusting for potential confounders. A mediation model tested whether family activity support mediated the relationship between parental PA and adolescents’ exercise cognition. Subgroup analyses explored heterogeneity across groups.

**Results:**

After adjusting for confounding variables, parental PA was positively associated with adolescents’ exercise cognition (*β* = 9.47 × 10^−2^, 95% CI: 8 × 10^−4^ to 1.1 × 10^−3^; *p* < 0.001). Family activity support was also positively associated with adolescents’ exercise cognition (*β* = 47.86 × 10^−2^, 95% CI: 1.045–1.118; *p* < 0.001). Family activity support mediated 49.05% of the total association of parental PA on adolescents’ exercise cognition, with a mediation effect size of 4.65 × 10^−2^, suggesting that higher parental PA was indirectly linked to improved adolescents’ exercise cognition through increased family activity support.

**Conclusion:**

The study found that both parental PA and family activity support were positively associated with adolescents’ exercise cognition, and family activity support mediated the relationship between parental PA and adolescents’ exercise cognition.

## Introduction

Exercise cognition in adolescents refers to an individual’s knowledge, beliefs, attitudes, and self-regulatory capacity regarding physical activity (PA), which encompasses executive functions (e.g., working memory and inhibitory control) and the decision-making process (1, 2). Identified risk factors included excessive electronic media use (3), physical inactivity (4), academic transition (5), and interpersonal influences such as family, peer, or societal pressures (5). Suboptimal exercise cognition constitutes a significant risk factor for physical inactivity and associated adverse health outcomes among youth, including increased risks for obesity, metabolic disorders, and poorer mental health trajectories (6). Therefore, enhancing exercise cognition among Chinese adolescents is crucial for addressing the increasing public health burden associated with sedentary lifestyles and promoting long-term health outcomes.

A previous study found that higher parental PA levels were significantly linked to increased PA levels in adolescent and may help mitigate the decline in adolescent PA (7). Supportive behaviors from parents, such as engaging in physical activities together and providing logistical support, may be key ways to enhance adolescents’ participation in sports and their exercise cognition (8). Potential mediating mechanisms may also include direct behavioral modeling (9), and the interaction with socioeconomic factors and overall parental lifestyle (10). Parental PA behavior serves as a cognitive model. By observing their parents’ emphasis on exercise—such as regular PA and sports-related responsibility—adolescents gradually develop the cognitive schema that exercise is essential for a healthy lifestyle. As a result, they are more likely to internalize these attitudes and enhance their exercise cognition (11). Therefore, examining the association between parental PA and adolescent exercise cognition is of particular interest in China, the world’s most populous country and second-largest economy.

Evidence suggests that inadequate support for family activities and reduced social support are strongly associated with poor cognitive and behavioral outcomes in adolescents (6). A supportive family activity environment, especially one marked by open communication with parents, can enhance adolescents’ perceived value of sports and increase their participation levels, which reflects more positive sports-related cognitive attitudes (12). The underlying mechanism may be that good family activity support helps to reduce depression in adolescents, and indirectly promotes exercise cognition by improving life satisfaction (13, 14). Limited studies only examined the association between parental PA and adolescent exercise cognition, and the association between family activity support and adolescent exercise cognition, respectively, but ignored the mediating role of family activity support between parental PA and adolescent exercise cognition. Based on the above research, we hypothesize that family activity support plays a partial mediating role in the relationship between parental PA and adolescents’ exercise cognition.

Therefore, this study aimed to explore the associations of parental PA and family activity support with adolescent exercise cognition. A mediation analysis framework was used to assess the potential mediating role of family activity support in the association between parental PA and adolescent exercise cognition. Such knowledge is necessary to develop targeted, family-based interventions to improve exercise cognition and promote healthy lifestyles in adolescents.

## Study methodology

### Data collection

**Figure 1:**
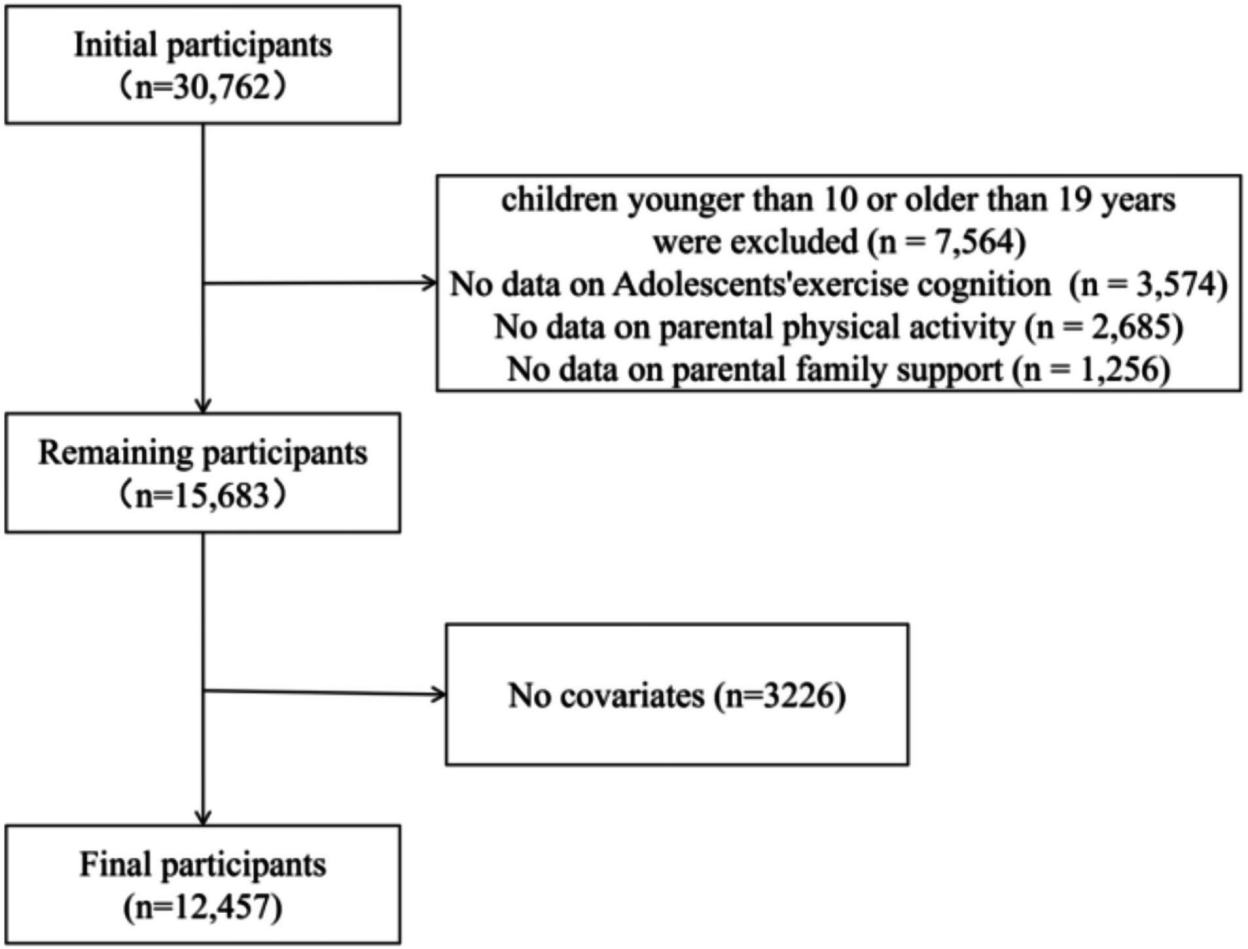
Flowchart of the participants selection process.

A cross-sectional design was used in this study. The survey was conducted in 15 provinces (autonomous regions and municipalities directly under the Central government) through the China Children and Adolescents Physical Education and Health Promotion Action School Construction Project of East China Normal University. The data collection of this study was conducted from April 2023 to September 2023, and the initial cohort included 30,762 respondents. For analytic rigor, exclusion criteria were adopted in sequence: children younger than 10 or older than 19 years were excluded (*n* = 7,564), those with incomplete exercise cognitive assessment for adolescents (*n* = 3,574), those without parental PA registration (*n* = 2,685), lack of information on family activity support (*n* = 1,256), and missing information on covariates (*n* = 3,226). This selection process resulted in the final analysis cohort of 12,457 participants, with complete exclusion paths shown in Figure 1.

### Assessments

#### Adolescents’ exercise cognition

The data on adolescents’ exercise cognition was collected in 2023. The exercise cognition of adolescents was evaluated through the exercise benefits and barriers scale-Chinese version (EBBS-CN), the use of which has been detailed in previous literature (15, 16). The EBBS-CN scale was scored using a four-point Likert scale, where 1 indicates strong agreement and 4 indicates strong disagreement. The 43-question questionnaire was designed to examine adolescents’ perceptions of the benefits and barriers of exercise. The perceived benefits section comprises 29 items, divided into five subscales: life improvement (8 questions), physical performance (8 questions), psychological state (6 questions), social interaction (4 questions), and preventive health (3 questions). Similarly, the perceived barriers section includes 14 items, divided into four subscales: exercise environment (6 questions), time consumption (3 questions), physical fatigue (3 questions), and family opposition (2 questions). To address the inherent issue of social desirability bias, we ensured participant anonymity throughout the questionnaire design, distribution, and collection phases.

### Parental physical activity

The data of Parental PA was collected in 2023. Parental PA levels were assessed using a validated short form of the International Physical Activity Questionnaire (IPAQ), a widely used tool that captures the frequency and duration of activities categorized as vigorous, moderate, and light intensity. Two key PA variables were derived: leisure-time physical activity (LPA), which includes activities aimed solely at exercise and recreation, and total physical activity (TPA), encompassing exercise, recreation, occupational demands, and other purposes. Participants’ daily durations for each type of activity were classified into four categories: 1 (≤0.5 h), 2 (0.5–2 h), 3 (2–4 h), and 4 (≥4 h). Weekly PA duration scores were obtained by multiplying the frequency of each activity by its corresponding daily duration index. These scores were then converted into metabolic equivalent (MET) values as follows: (1) LPA MET score = 8.0 × weekly leisure vigorous activity score + 4.0 × weekly leisure moderate activity score + 3.3 × weekly leisure walking score; and (2) TPA MET score = 8.0 × weekly total vigorous activity score + 4.0 × weekly total moderate activity score + 3.3 × weekly total walking score. The TPA and LPA scores were then divided into two categories: those with a score of zero (no reported PA) and those with a score greater than zero (indicating participation in PA). Among participants who engaged in PA, further classification into low, moderate, and high activity levels was made based on tertiles of their TPA/LPA MET scores (17). To minimize social desirability bias, we ensured participant anonymity during questionnaire design, distribution, and collection.

### Family activity support

The data on family activity support was collected in 2023. Family Activity Support was assessed by the Activity Support Scale for Multiple Groups-Chinese Version (ACTS-CN) (18). The ACTS-CN was completed by adolescents independently. The scale examined the family support for adolescent PA through parents’ PA behavior and their attitudes and influences on adolescent PA behavior.

The ACTS-CN was divided into two scales, the mother volume and the father volume. The mother and father volume of ACTS-CN had 9 questions respectively, which were designed with a 4-point Likert scale and divided into two sub-dimensions: activity supportive behaviors and attitudes and activity behavior restrictions. The sub-dimension of activity support behavior and attitude included 6 questions, all of which were positive questions on a 4-point Likert scale, with 1 indicating “strongly disagree” and 4 indicating “strongly agree.” The number corresponding to the choice was the score of the topic. The questions included parents’ exercise habits, reminding, accompanying, paying attention to their children’s exercise, providing convenience for their children’s exercise venues, supporting their children to participate in clubs/training classes, etc. The activity behavior restriction sub-dimension included three questions, all of which were negative questions on a 4-point Likert scale, with 1 indicating “strongly disagree” and 4 indicating “strongly agree.” The number 5 minus the corresponding option was the score of the item. If you choose “1” = very disagree in question 1, then the score of this question is recorded as “4 points” = 5–1. Questions included whether children should be allowed to watch TV, use computers and play video games without restrictions. The final score of ACTS-CN was the sum of the scores (including positive and negative scores) of all questions about parents. To reduce the influence of social desirability bias, participant identities were kept anonymous throughout the stages of questionnaire development, administration, and data collection.

### Validity and reliability of the questionnaire

The Kaiser–Meyer–Olkin (KMO) and Bartlett sphericity tests were used to analyze the validity of EBBS, IPAQ and ACTS-CN, and the results are shown in Table 1. The KMO of exercise cognition in adolescents was 0.982, the KMO of parental PA was 0.618, and the KMO of family activity support was 0.888. The KMO of each variable was greater than 0.6, and the *p* value of Bartlett’s sphericity test was less than 0.001, indicating that the validity of all questionnaires was acceptable.

**Table 1:** Validity analysis.

Variables	Validity		
Adolescents’ exercise cognition	KMO		0.982
	BTS	*χ* ^2^	497,977.370
		df	903
		*P*	<0.001
Parental physical activity	KMO		0.618
	BTS	*χ* ^2^	130,634.797
		df	231
		*P*	<0.001
Family activity support	KMO		0.888
	BTS	*χ* ^2^	174,441.910
		df	153
		*P*	<0.001

KMO, Kaiser–Meyer–Olkin. BTS, Bartlett’s Test of Sphericity. *χ*^2^, Approx. Chi-Square; df, Degrees of Freedom.

The reliability tests of all questionnaires are shown in Table 2, and Cronbach’s *α* test was performed on the questionnaires. The Cronbach’s α coefficient of students’ exercise cognition, parents’ PA and family activity support was 0.957, 0.657 and 0.840, respectively. The Cronbach’s α coefficients of all the questionnaire scales were greater than 0.6, indicating that the reliability of the questionnaire was acceptable.

**Table 2:** Reliability analysis.

Variables	Number of items	Cronbach’s *α*
Adolescents’ exercise cognition	43	0.957
Parental physical activity	22	0.657
Family activity support	18	0.840

### Measurement model assessment: confirmatory factor analysis

AMOS 24.0 was used to perform confirmatory factor analysis. Model fit indices were: Chi-square to degrees of freedom ratio (*χ*^2^/df) = 3.861, root mean square error of approximation (RMSEA) = 0.074, comparative fit index (CFI) = 0.919, goodness-of-fit index (GFI) = 0.908, incremental fit index (IFI) = 0.919, normed fit index (NFI) = 0.918, indicating acceptable model fit. The model effectively reflects the structural relationships between observed and latent variables.

As shown in Supplementary Table 1, standardized factor loadings for the latent variables—parental PA, family activity support, and adolescent exercise cognition—were all above 0.5, demonstrating adequate item representation. Average variance extracted (AVE) exceeded 0.5 and composite reliability (CR) exceeded 0.7 for each variable, supporting good convergent validity and internal consistency.

As shown in Supplementary Table 2, the absolute correlation coefficients among parental PA, family activity support, and adolescent exercise cognition are all less than the square root of their respective AVE values, indicating adequate discriminant validity. These variables are moderately associated yet distinct, supporting the scale’s overall discriminant validity.

### Control variables

The control variables at baseline survey included the adolescent’s gender (divided into male/female), parental education level (divided into never attended school, primary school, junior high school, senior high school/vocational high school/junior college/bachelor’s degree, master’s degree or above), adolescent’s school period (primary school/junior high school/senior high school), and parental body mass index (BMI). Parental height and weight were collected with the use of standardized techniques to calculate BMI. According to World Health Organization guidelines, parental BMI was categorized into four categories: underweight (BMI < 18.5 kg/m^2^), normal (18.5–24.9 kg/m^2^), overweight (25–29.9 kg/m^2^), and obese (≥ 30 kg/m^2^).

### Statistical methods

All statistical analyses in this cross-sectional study were performed with the use of R, version 4.3.3. Descriptive statistics of sample characteristics are presented as means and standard deviations for continuous variables and as frequencies and percentages for categorical variables. Shapiro–Wilk test was used for normality of continuous variables. Parental PA was natural log-transformed. The relationship between the main variables was analyzed using Spearman’s rank correlation. Multiple linear regression quantified the associations between parental PA, family activity support, and adolescents’ exercise cognition using standardized *β* coefficients. Two adjusted models were identified: (1) unadjusted model. (2) Adjusted models: adolescent gender, adolescent schooling period, parental education level and parental BMI. Stratified subgroup analyses were performed to test for potential effect heterogeneity. Subsequently, the Baron and Kenny mediation model was used to examine the mediating effect of family activity support on parental PA and adolescents’ exercise cognition (19). The mediating effect was quantified by calculating the proportion of variance explained by the mediating variable, and its significance was assessed using bootstrap resampling with 1,000 replications. *p* < 0.05 was considered statistically significant.

## Result

### Characteristics of the study participants

Table 3 presents the basic characteristics of the research sample, which consisted of 12,457 participants. Significant differences were observed in exercise cognition scores among adolescents based on grade level, gender, and parental educational attainment (*p* < 0.05). Exercise cognition scores decreased with increasing grade level, with the highest scores found in grades 4–6, followed by grades 7–9, and the lowest in grades 10–12. Male adolescents demonstrated significantly higher exercise cognition scores compared to female adolescents. Furthermore, adolescents whose parents had attained a high school, vocational high school, technical secondary school, or college/bachelor’s degree level of education exhibited significantly higher exercise cognition scores than those whose parents had completed only primary or junior high school education.

**Table 3:** Participants’ basic characteristics (*N* = 12,457).

Variables		Adolescents’ exercise cognition	Family activity support	Parental physical activity
Adolescents’ grade (Mean ± SD)	① Grades 4–6	132.34 ± 18.46	51.83 ± 7.72	2851.38 ± 1828.04
	② Grades 7–9	128.18 ± 17.84	48.34 ± 7.95	2952.56 ± 1818.52
	③ Grades 10–12	127.03 ± 17.97	46.42 ± 7.78	2969.96 ± 1810.63
	*F*	99.018	469.16	5.396
	*p*	<0.001	<0.001	0.009
	LSD/Games–Howell	① > ② > ③	① > ② > ③	②③ > ①a
Adolescents’ sex (Mean ± SD)	Male	131.73 ± 18.55	49.87 ± 8.14	2875.91 ± 1831.92
	Female	128.23 ± 17.87	49.57 ± 8.06	2937.94 ± 1812.23
	*t*	10.728	2.023	−1.899
	*P*	<0.001	0.043	0.058
Parental BMI (Mean ± SD)	① Underweight	129.45 ± 17.95	50.07 ± 8.41	2715.21 ± 1858.83
	② Normal Weight	130.33 ± 18.20	49.88 ± 8.05	2908.93 ± 1804.27
	③ Overweight	129.05 ± 18.60	49.61 ± 7.94	2983.11 ± 1811.87
	④ Obese	129.05 ± 18.45	48.88 ± 8.50	2838.26 ± 1914.90
	*F*	2.266	6.303	5.457
	*P*	0.079	<0.001	<0.001
	LSD/Games–Howell	–	①②③ > ④a	②③ > ①
Parental education level (Mean ± SD)	① Uneducated	123.09 ± 20.64	49.17 ± 8.20	2081.93 ± 2118.94
	② Elementary school	127.63 ± 17.87	48.92 ± 7.97	2687.65 ± 1997.33
	③ Junior high school	128.22 ± 17.22	48.65 ± 7.82	2991.32 ± 1891.77
	④ High school	130.53 ± 17.82	49.78 ± 7.96	3082.53 ± 1810.73
	⑤ Undergraduate	131.23 ± 19.13	50.47 ± 8.29	2759.02 ± 1742.99
	⑥ Master’s degree or above	131.44 ± 22.10	51.96 ± 8.66	2513.83 ± 1686.53
	*F*	14.267	25.331	19.056
	*P*	<0.001	<0.001	<0.001
	LSD/Games–Howell	④⑤ > ②③	④ > ③ ⑤⑥ > ②③④	③④ > ②⑤⑥

LSD test was used for post hoc analysis, while Games–Howell test was used for statistical analysis in other cases.

There were significant differences in family activity support scores based on students’ grade level, gender, and parents’ BMI and education level (*p* < 0.05). Specifically, family activity support scores decreased with higher grade levels, with the highest scores observed in grades 4–6, followed by grades 7–9, and the lowest in grades 10–12. Boys received significantly higher family activity support scores than girls. Among parents, those who were overweight, normal weight, or underweight provided significantly higher support than those classified as obese. In terms of parental education level, family activity support scores were highest for parents with a university degree (bachelor’s, master’s, or higher), followed by those with a high school or vocational/technical secondary school education, while the lowest scores were observed among parents with only a primary or junior high school education.

There were significant differences in parents’ PA levels based on their children’s grade levels, as well as parents’ BMI and educational attainment (*p* < 0.05). Parents of children in grades 7–9 and 10–12 exhibited significantly higher PA levels compared to parents of children in grades 4–6. Additionally, parents with normal and overweight BMI demonstrated significantly higher PA levels than those with underweight BMI. With regard to educational attainment, parents who completed junior high school or senior high school/vocational high school/secondary technical school showed higher PA levels than those with only a primary school education, while parents with university/bachelor’s or postgraduate degrees had the lowest levels of PA.

Moreover, the analysis of baseline characteristics of included and excluded participants is shown in Supplementary Table 3. Significant differences were observed between the participant and exclusion groups in terms of adolescents’ sex, grade, and parental education (all *p* < 0.001).

### The relationship between important variables

Table 4 presents the associations among parents’ PA, family activity support, and adolescents’ exercise cognition. This analysis examined the association between parents’ PA and adolescents’ exercise cognition among 12,610 participants. The unadjusted analysis revealed a statistically significant positive association between parents’ PA and adolescents’ exercise cognition, with a *β* of 8.70 × 10^−2^ (95% CI: 7 × 10^−4^ to 1 × 10^−3^; *p* < 0.001). In the fully adjusted model, *β* slightly increased (*β* = 9.47 × 10^−2^, 95% CI: 8 × 10^−4^ to 1.1 × 10^−3^; *p* < 0.001). This consistency indicates that covariate adjustment had a minimal impact on the positive association between parents’ PA and adolescents’ exercise cognition.

**Table 4:** Association of parental physical activity and family activity support with exercise cognition in adolescents (*n* = 12,457).

Variables	Unadjusted		Adjusted	
	*β* (95% CI)	*P*	*β* (95% CI)	*P*
Parental physical activity	8.70 × 10^−2^ (7 × 10^−4^ to 1.0 × 10^−3^)	<0.001	9.47 × 10^−2^ (8 × 10^−4^ to 1.1 × 10^−3^)	<0.001
Family activity support	48.01 × 10^−2^ (1.050–1.120)	<0.001	47.86 × 10^−2^ (1.045–1.118)	<0.001

Adjusted for adolescent gender, grade level, parental BMI, and parental education level. *β*, Standardized regression coefficients; 95% CI, 95% confidence interval.

Furthermore, the unadjusted analysis revealed a statistically significant positive association between family activity support and adolescents’ exercise cognition (*β* = 48.08 × 10^−2^, 95% CI: 1.050–1.198; *p* < 0.001). In the fully adjusted model, the β value slightly decreased (*β* = 47.86 × 10^−2^, 95% CI: 1.045–1.118; *p* < 0.001), indicating that the association remained robust even after controlling for potential confounders.

Supplementary Table 4 presents subgroup analyses of the association between parental PA and family activity support, showing consistent results across all subgroups (*p* > 0.05 for interaction). Supplementary Table 5 displays similar subgroup analyses for the association between parental PA and adolescents’ exercise cognition, also showing consistency across subgroups (*p* > 0.05 for interaction). Table 5 further highlights that adolescent grade (*p* < 0.001 for interaction) and gender (*p* < 0.001 for interaction) significantly modified the association between family activity support and adolescents’ exercise cognition.

**Table 5:** Subgroup analysis of the relationship between family activity support and exercise cognition in adolescents.

Subgroup	*n*	Exercise cognition Mean ± SD	*β* (95% CI)	*P* for interaction
Parental education level				0.241
Never	23	19.78 ± 3.21	0.7 (0.35, 1.05)	
Primary school	530	20.08 ± 2.88	0.49 (0.42, 0.57)	
Middle school	3,527	20.52 ± 3.13	0.44 (0.41, 0.47)	
High school	3,490	20.17 ± 3.23	0.46 (0.43, 0.49)	
Undergraduate	4,635	19.60 ± 2.94	0.51 (0.49, 0.54)	
Master’s degree or above	252	20.00 ± 3.36	0.6 (0.48, 0.72)	
Parental BMI				0.777
Under weight	843	20.25 ± 3.39	0.49 (0.44, 0.55)	
Normal weight	7,354	20.22 ± 3.24	0.48 (0.46, 0.50)	
Over weight	2,970	20.03 ± 3.01	0.47 (0.44, 0.50)	
Obese	1,290	19.62 ± 2.89	0.48 (0.43, 0.52)	
Adolescents’ grade				<0.001
Primary school	6,056	20.09 ± 3.34	0.57 (0.55, 0.60)	
Middle school	4,386	20.01 ± 3.24	0.43 (0.40, 0.45)	
High school	2015	19.96 ± 2.81	0.33 (0.29, 0.37)	
Adolescents’ gender				<0.001
Male	6,376	20.05 ± 2.96	0.44 (0.42, 0.46)	
Female	6,081	19.99 ± 3.29	0.52 (0.50, 0.54)	

Adjusted for adolescent gender, grade level, parental BMI, and parental education level. *β*, Standardized regression coefficients; 95% CI, 95% confidence interval; BMI, body mass index.

### Family activity support mediates the relationship between parental physical activity and adolescents’ exercise cognition

Table 6 presents the interrelationships between baseline parental PA, family activity support, and adolescents’ exercise cognition. The analysis revealed significant positive associations among these variables: parental PA was positively associated with adolescents’ exercise cognition (*r* = 0.087, *p* < 0.001), and even more so with family activity support (*r* = 0.087, *p* < 0.001). Notably, the association between family activity support and adolescents’ exercise cognition was substantially stronger (*r* = 0.480, *p* < 0.001).

**Table 6:** Association analysis of parental physical activity, family activity support and exercise cognition in adolescents.

Variables	Parental physical activity	Family activity support	Adolescents’ exercise cognition
Parental physical activity	1		
Family activity support	0.087^***^	1	
Adolescents’ exercise cognition	0.087^***^	0.480^***^	1

*** *p* < 0.001.

**Figure 2:**
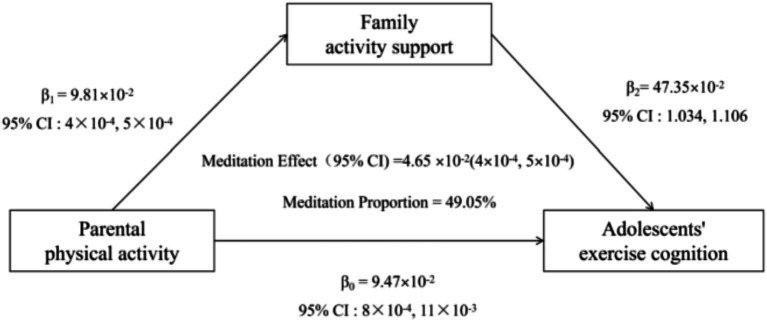
The conceptional framework of the mediation models. *β*_0_ was the total effect of parental physical activity on adolescents’ exercise cognition; *β*_1_ represents the effect of parental physical activity on family activity support; *β*_2_ represents the effect of Family activity support on adolescents’ exercise cognition. The mediation effect was computed as the product of “*β*_1_” and “*β*_2_” (*β*_1_ × *β*_2_), and the mediation proportion was calculated as the ratio of the mediation effect product to total effects [(*β*_1_ × *β*_2_)/*β*_0_].

Bootstrap analysis determined the baseline total effect of parental PA on exercise cognition in adolescents (*β*_0_ = 9.47 × 10^−2^, 95% CI: 8 × 10^−4^ to 1.1 × 10^−3^). Family activity support played a mediating role in the association between parental PA and adolescents’ exercise cognition. The mediating effect size was 4.65 × 10^−2^ (95%CI = 4 × 10^−4^ to 5 × 10^−4^), explaining 49.05% of the total effect variation. The mediated pathway is shown in Figure 2.

## Discussion

This study, conducted across 15 provinces and municipalities in China in 2023, included a final sample of 12,457 participants and investigated the association between parental PA, family activity support, and adolescents’ exercise cognition. The results indicated that both parental PA and family activity support were positively associated with adolescents’ exercise cognition. Moreover, family activity support partially mediated the relationship between parental PA and adolescents’ exercise cognition. The grade and gender of adolescents significantly influenced the association between family activity support and adolescents’ exercise cognition, thereby supporting the initial hypothesis of this study.

Previous research indicates that parental PA, particularly regular exercise, can have significant intergenerational effects on offspring, primarily manifested as improvements in cognitive function (20). Using a mouse model to simulate human conditions and found that regular exercise interventions in parents led to enhanced cognitive abilities in their direct offspring, including improvements in both spatial and non-spatial memory. This study, which involved surveys of parents and their adolescent children, demonstrated that, after adjusting for relevant confounding factors, a positive association existed between parental PA and adolescents’ exercise cognition (*β* = 8.70 × 10^−2^, 95% CI: 7 × 10^−4^ to 1 × 10^−3^, *p* < 0.001). The mechanisms linking parental PA to adolescents’ exercise cognition involve a complex, multi-level research area. Parental PA serves as a direct role model, which may be associated with adolescents’ perceptions and attitudes toward exercise. When adolescents observe their parents engaging in PA, they may be more likely to internalize the value of exercise and develop greater self-efficacy, leading to increased time spent on PA (21, 22). Regular PA could enhance adolescents’ exercise cognition by increasing levels of neurotrophic factors such as brain derived neurotrophic factor (BDNF) and vascular endothelial growth factor (VEGF), particularly in the functional performance of the frontal and temporal lobes (23). Secondly, parents’ positive emotions during PA can reduce their children’s anxiety. Evidence shows that when parents engage in moderate to vigorous activity at least three times a week, it indirectly enhances children’s cognitive abilities by lowering anxiety (24).

This study shows a positive association between parental PA and family activity support. Higher levels of parental PA significantly are associated with a more supportive family environment, which may include family atmosphere, resource allocation, values, and behavioral patterns through interconnected mechanisms, thereby being associated with higher levels of family activity support. Firstly, adolescents, especially younger children, primarily learn by observing and imitating significant adults in their lives, such as their parents. When parents regularly engage in PA, children directly perceive it as a normal, valuable, and even enjoyable part of daily life (25). Secondly, parental consistency in PA sends a powerful message: health matters, the body requires care, and PA is essential to maintaining it. Over time, families may develop a shared identity—“we are an active, sports-loving family”—which can strengthen motivation to participate (26). Furthermore, parents who are physically active are more likely to schedule family activities like weekend hikes, cycling, swimming, or playing sports, rather than letting screen time dominate. They also tend to invest in sports equipment or enroll children in sports programs, supporting greater family participation in PA.

Previous research shows that when parents encourage or join their children in aerobic activities like running and swimming, they improve their children’s aerobic fitness, which enhances neural signal transmission efficiency and, in turn, boosts working memory and cognitive flexibility (2). Furthermore, a cross-sectional study shows that teenagers’ time spent on electronic media is negatively linked to cognitive function. If parents reduce sedentary behavior and encourage more family PA, they might be able to cut children’s screen time by an average of 10 h per week, thus potentially freeing up attention resources and supporting the development of higher-order cognitive abilities (3). Mediation analysis showed that family activity support mediated the link between parental PA and adolescents’ exercise cognition, with an effect size of 4.65 × 10^−2^ (95% CI = 4 × 10^−4^ to 5 × 10^−4^), and was positively associated with adolescents’ exercise cognition. The underlying mechanisms may include the following: Firstly, when families provide support—such as jointly planning exercise time and supplying equipment—adolescents are more likely to overcome objective barriers to PA, such as time conflicts and lack of resources, thereby strengthening their perception of exercise value and willingness to maintain it (27). Secondly, family activity support could enhance adolescents’ subjective ability to try new sports by reducing psychological pressure, such as frustration, thus promoting deeper exercise cognition (28, 29). For example, adolescents in the high family activity support group exhibited fewer depressive symptoms, and this positive psychological state may foster openness to PA (30). Thirdly, adolescents may be more likely to explore new activities and accumulate exercise knowledge through practice when families foster a non-critical environment—such as encouraging experimentation rather than focusing on outcomes—whereas family conflict may inhibit exploration motivation (31, 32). Finally, a daily 50-min moderate-intensity exercise session performed together by parents and children has been linked to improved cortisol rhythm stability by 31%, which in turn may optimized motor cognitive performance (33, 34).

The finding that family activity support was estimated to account for nearly half (49.05%) of the total association between parental PA and adolescent exercise cognition highlights a potential pathway for behavioral transmission in families that goes beyond mere imitation. This strong mediation is best understood through Social Cognitive Theory (SCT) and Family Systems Theory (35–37). SCT identifies parents as key role models whose PA behaviors are observed and learned by adolescents. Our results suggest this modeling effect is amplified primarily when embedded in a supportive family environment. Parental PA does not influence adolescents only through direct observation—it actively shapes a family culture of support, including practical help, encouragement, and shared activities, which becomes the most immediate and powerful influence on adolescent exercise cognition. This reflects SCT’s triadic reciprocity, where personal, behavioral, and environmental factors interact (35). Furthermore, family Systems Theory further explains the power of this environment. It views the family as an interconnected unit where one member’s behavior—such as parental PA—can disrupt homeostasis and trigger system-wide changes. A physically active parent acts as an agent of systemic change, reshaping family routines, values, and emotional connections around health. Thus, family activity support is not just a set of behaviors but an emergent feature of an adapting family system (36). The 49.05% indirect effect quantifies the proportion of the observed association between parental PA and adolescent exercise cognition that operated through family activity support in our model.

However, these findings must be interpreted within the limits of the cross-sectional design. While the observed associations and the proposed mediation by family activity support are theoretically grounded and statistically significant, they do not establish causality. The study’s main contribution is identifying a plausible mechanism and generating strong hypotheses. The mediation model provides a necessary foundation for future longitudinal studies to confirm temporal sequences and for family-based interventions to test causal relationships.

Based on this study’s findings, public health interventions and policies aiming to improve adolescents’ exercise cognition and PA should adopt a dual focus: (1) Directly promoting parental PA through community programs and awareness campaigns, as active parents model positive behaviors, and (2) Actively fostering family activity support systems. Policies should provide resources and guidance to help families engage in PA together, offer logistical support (e.g., access to facilities, time management), and create supportive home environments. This family-centered approach, particularly targeting higher-grade adolescents and girls where support’s impact is most significant, can leverage the mediating role of family support to amplify the benefits of parental PA on adolescent health cognition and long-term outcomes.

Future studies should employ longitudinal designs to establish temporal precedence and causal relationships between parental PA, family activity support, and adolescents’ exercise cognition. Conduct multi-wave follow-up surveys to examine whether changes in parents’ PA precede changes in family activity support and subsequently influence the transformation of adolescents’ exercise cognition. Experimental or quasi-experimental interventions targeting parental PA and family support behaviors are needed to confirm mediation effects. Design family-based randomized controlled trials to verify the causal mediation path found in this study by intervening in parents’ PA behaviors or family support skills. Research should further explore potential moderators (e.g., socioeconomic status, cultural context) and additional mediating pathways, such as biological mechanisms (e.g., neurotrophic factors) or psychosocial factors (e.g., family cohesion, adolescent self-efficacy), to deepen understanding of the underlying processes. Multimethod approaches integrating objective PA measures are also recommended.

### Advantages and limitations

This study has some advantages. Firstly, the study utilized a large-scale, nationally representative sample (12,457 participants across 15 Chinese provinces/regions), enhancing statistical power and generalizability of findings to diverse adolescent populations in China. Secondly, the research employed rigorous, validated instruments (EBBS for exercise cognition, IPAQ for parental PA, ACTS-CN for family support) with demonstrated reliability (Cronbach’s *α*: 0.657–0.957) and acceptable validity (KMO > 0.6), ensuring robust measurement of core constructs. Thirdly, the analysis adopted a comprehensive methodological approach, including mediation modeling (Baron and Kenny), stratification, sensitivity analyses (e.g., restricted cubic splines), and control for key confounders (e.g., gender, parental BMI/education), which strengthened the robustness of the observed associations and identified nuanced pathways (e.g., family support as a mediator). However, this study has several limitations that should be acknowledged. Firstly, this cross-sectional design cannot establish causal relationships or temporal order. Due to data synchronicity, mediation analysis (e.g., Baron and Kenny’s framework) is inherently limited in inferring the sequence of “parents’ PA → family activity support → adolescents’ exercise cognition.” While theoretically plausible, this path may be subject to reverse causality. Secondly, self-reported measures (e.g., parental PA, family support) are susceptible to recall/social desirability bias, and objective PA monitoring (e.g., accelerometers) was not used. Thirdly, despite adjusting for several covariates, residual confounding from unmeasured variables may bias the observed associations and mediation effect. For example, parents’ attitudes toward sports and healthy living—a potential common cause—could influence both their PA levels and their provision of family support. Similarly, adolescents’ baseline personality traits (e.g., conscientiousness, openness) may affect their interest in PA and shape how they perceive or elicit parental support. Peer influence, a strong adolescent driver, could also independently affect exercise cognition and behavior, potentially confounding the role of family systems. Omitting these and other shared factors may lead to overestimating the mediation effect by failing to account for third variables that influence both the mediator and outcome. Fourthly, 18,305 participants were excluded due to missing data or incomplete evaluations. The included and excluded groups differed significantly in adolescent gender, grade, and parental education, which may introduce selection bias and limit the generalizability of the findings. Fifthly, the focus on Chinese adolescents restricts generalizability to other cultural/socioeconomic context. Sixthly, peer/school influences on exercise cognition were not assessed, omitting key ecological systems beyond the family. Seventhly, the lack of longitudinal data hinders examination of how parental PA/family support impacts exercise cognition trajectories during critical developmental stages. Eighthly, social desirability bias is an inherent limitation of self-reported data. Students may have overreported family activity support and cognitive aspects of exercise, which could artificially inflate the observed correlations and lead to an overestimation of the mediation effect. Finally, biological mechanisms (e.g., neurotrophic factors like BDNF) linking PA to cognition were inferred but not empirically measured.

## Conclusion

This study demonstrates that parental PA and family activity support are positively associated with adolescents’ exercise cognition. The mediation analysis suggests that family activity support may serve as a key intermediary in this relationship. These findings highlight a potential psychological-social pathway and provide a foundation for testable hypotheses regarding the supportive role of family activities in longitudinal or intervention studies.

## Data Availability

The original contributions presented in the study are included in the article/Supplementary material, further inquiries can be directed to the corresponding author.
