# Supplementary material for: Relationship between parental physical activity and adolescents’ exercise cognition: the mediating role of family activity support
Source: Front Public Health. 2025 Dec 2;13:1685991. doi: 10.3389/fpubh.2025.1685991 (PMC12705581; doi:10.3389/fpubh.2025.1685991)
Supplement: Supplementary file 1 [file Table_1.DOCX]

Supplementary Table 1. Confirmatory factor analysis results: factor loadings, reliability, and validity metrics

| **Pathway** | **Standardized regression coefficient** | **Unstandardized regression coefficients** | **AVE** | **CR** | **p** |
| --- | --- | --- | --- | --- | --- |
| Support→Mother1 | 0.758 | 1 | 0.637 | 0.875 |  |
| Support→Mother2 | 0.766 | 1.014 |  |  | <0.001 |
| Support→Father1 | 0.845 | 1.175 |  |  | <0.001 |
| Support→Father2 | 0.821 | 1.146 |  |  | <0.001 |
| Cognition→Cognition1 | 0.904 | 1 | 0.798 | 0.940 |  |
| Cognition→Cognition2 | 0.933 | 1.069 |  |  | <0.001 |
| Cognition→Cognition3 | 0.949 | 1.162 |  |  | <0.001 |
| Cognition→Cognition4 | 0.778 | 1.163 |  |  | <0.001 |
| Parental→PA1 | 0.506 | 1 | 0.540 | 0.766 |  |
| Parental→PA2 | 0.972 | 1.813 |  |  | <0.001 |
| Parental→PA3 | 0.648 | 17.14 |  |  | <0.001 |
| Abbreviation:AVE, Average Variance Extracted. CR, Composite Reliability. | | | | | |
