# Supplementary material for: Relationship between parental physical activity and adolescents’ exercise cognition: the mediating role of family activity support
Source: Front Public Health. 2025 Dec 2;13:1685991. doi: 10.3389/fpubh.2025.1685991 (PMC12705581; doi:10.3389/fpubh.2025.1685991)
Supplement: Supplementary file 2 [file Table_2.DOCX]

Supplementary Table 2. Discriminant Validity

|  | Family activity support | Exercise cognition | Parental PA |
| --- | --- | --- | --- |
| Family activity support | **0.798** |  |  |
| Exercise cognition | 0.496^***^ | **0.894** |  |
| Parental PA | 0.115^***^ | 0.100^***^ | **0.735** |
| Abbreviation:PA, Physical activity. | | | |
