# Supplementary material for: Relationship between parental physical activity and adolescents’ exercise cognition: the mediating role of family activity support
Source: Front Public Health. 2025 Dec 2;13:1685991. doi: 10.3389/fpubh.2025.1685991 (PMC12705581; doi:10.3389/fpubh.2025.1685991)
Supplement: Supplementary file 3 [file Table_3.DOCX]

Supplementary Table 3: Demographic comparison between the exclusion and adoption groups

| **Variables** | | **Exclusion group**  **(n=18305)** | **Adopting group**  **(n=12457)** | **X^2^** | **p** |
| --- | --- | --- | --- | --- | --- |
| Gender | Male | 9807(53.6) | 6376(51.2) | 17.003 | <0.001 |
|  | Female | 8498(46.4) | 6081(48.8) |  |  |
| Grade level | Grades 1 to 3 | 7564(41.3) | 0(0.0) | 8293.147 | <0.001 |
|  | Grades 4 to 6 | 3564(19.5) | 6056(48.6) |  |  |
|  | Grades 7 to 9 | 3125(17.1) | 4386(35.2) |  |  |
|  | Grades 10 to 12 | 4052(22.1) | 2015(16.2) |  |  |
| BMI level | Underweight | 1161(6.3) | 843(6.8) | 6.122 | 0.106 |
|  | Normal weight | 10672(58.3) | 7354(59.0) |  |  |
|  | Overweight | 4469(24.4) | 2970(23.8) |  |  |
|  | Obesity | 2003(10.9) | 1290(10.4) |  |  |
| Educational level | Never having attended school | 52(0.3) | 23(0.2) | 254.948 | <0.001 |
|  | Primary School | 613(3.3) | 530(4.3) |  |  |
|  | Middle School | 4269(23.3) | 3527(28.3) |  |  |
|  | High School / Vocational School / Technical Secondary School | 4552(24.9) | 3490(28.0) |  |  |
|  | University / Undergraduate Education | 8321(45.5) | 4635(37.2) |  |  |
|  | Master's degree or above | 498(2.7) | 252(2.0) |  |  |
| Abbreviation:BMI, Body mass index. X^2^, Chi-square statistic. | | | | | |
