# Supplementary material for: Relationship between parental physical activity and adolescents’ exercise cognition: the mediating role of family activity support
Source: Front Public Health. 2025 Dec 2;13:1685991. doi: 10.3389/fpubh.2025.1685991 (PMC12705581; doi:10.3389/fpubh.2025.1685991)
Supplement: Supplementary file 4 [file Table_4.DOCX]

| Supplementary Table 4. Subgroup analysis of the relationship between parental physical activity and family activity support | | | | |
| --- | --- | --- | --- | --- |
| Subgroup | n | Family activity support (Mean±SD) | β (95% CI) | P for interaction |
| Parental Education Level |  |  |  | 0.493 |
| Never | 23 | 30.07±4.78 | 0.18（-0.24, 0.59） |  |
| Primary school | 530 | 30.26±5.01 | 0.05（-0.03, 0.13） |  |
| Middle school | 3527 | 29.52±4.77 | 0.08（0.05, 0.11） |  |
| High school | 3490 | 29.51±5.15 | 0.1（0.07, 0.13） |  |
| Undergraduate | 4635 | 29.97±5.16 | 0.1（0.07, 0.13） |  |
| Master's degree or above | 252 | 29.42±4.75 | 0.19（0.04, 0.33） |  |
| Parental BMI |  |  |  | 0.25 |
| Under weight | 843 | 30.27±5.44 | 0.12（0.05, 0.19） |  |
| Normal weight | 7354 | 29.02±5.05 | 0.08（0.06, 0.10） |  |
| Over weight | 2970 | 30.30±4.29 | 0.11（0.07, 0.14） |  |
| Obese | 1290 | 29.55±4.89 | 0.06（0.01, 0.12） |  |
| Adolescents' grade |  |  |  | 0.208 |
| Primary school | 6056 | 29.74±5.13 | 0.11（0.08, 0.13） |  |
| Middle school | 4386 | 29.62±4.78 | 0.08（0.06, 0.11） |  |
| High school | 2015 | 30.01±4.86 | 0.05（0.01, 0.10） |  |
| Adolescents' gender |  |  |  | 0.887 |
| Male | 6376 | 29.77±4.83 | 0.08（0.05, 0.10） |  |
| Female | 6081 | 29.82±5.02 | 0.09（0.06, 0.11） |  |
| Adjusted for adolescent gender, grade level, parental BMI, and parental education level. Abbreviations: β, Standardized regression coefficients; 95% CI, 95% confidence interval; BMI, body mass index. | | | | |
