# Supplementary material for: Relationship between parental physical activity and adolescents’ exercise cognition: the mediating role of family activity support
Source: Front Public Health. 2025 Dec 2;13:1685991. doi: 10.3389/fpubh.2025.1685991 (PMC12705581; doi:10.3389/fpubh.2025.1685991)
Supplement: Supplementary file 5 [file Table_5.DOCX]

| Supplementary Table 5. Subgroup analysis of the association between parental physical activity  and adolescents' exercise cognition | | | | |
| --- | --- | --- | --- | --- |
| Subgroup | n | Adolescents' exercise cognition (Mean±SD) | β (95% CI) | P for interaction |
| Parental Education Level |  |  |  | 0.349 |
| Never | 23 | 19.78±3.21 | 0.3（-0.12, 0.72） |  |
| Primary school | 530 | 20.08±2.88 | 0.13（0.05, 0.20） |  |
| Middle school | 3527 | 20.52±3.13 | 0.07（0.04, 0.10） |  |
| High school | 3490 | 20.17±3.23 | 0.1（0.07, 0.14） |  |
| Undergraduate | 4635 | 19.60±2.94 | 0.08（0.05, 0.11） |  |
| Master's degree or above | 252 | 20.00±3.36 | 0.15（-0.01, 0.31） |  |
| Parental BMI |  |  |  | 0.707 |
| Under weight | 843 | 20.25±3.39 | 0.08（0.01, 0.14） |  |
| Normal weight | 7354 | 20.22±3.24 | 0.09（0.07, 0.11） |  |
| Over weight | 2970 | 20.03±3.01 | 0.09（0.05, 0.12） |  |
| Obese | 1290 | 19.62±2.89 | 0.08（0.03, 0.13） |  |
| Adolescents' grade |  |  |  | 0.146 |
| Primary school | 6056 | 20.09±3.34 | 0.1（0.08, 0.13） |  |
| Middle school | 4386 | 20.01±3.24 | 0.08（0.05, 0.10） |  |
| High school | 2015 | 19.96±2.81 | 0.09（0.04, 0.13） |  |
| Adolescents' gender |  |  |  | 0.07 |
| Male | 6376 | 20.05±2.96 | 0.1（0.08, 0.13） |  |
| Female | 6081 | 19.99±3.29 | 0.07（0.05, 0.10） |  |
| Adjusted for adolescent gender, grade level, parental BMI, and parental education level. Abbreviations: β, Standardized regression coefficients; 95% CI, 95% confidence interval; BMI, body mass index. | | | | |
